# N^6^-methyladenosine (m^6^A) writer METTL5 represses the ferroptosis and antitumor immunity of gastric cancer

**DOI:** 10.1038/s41420-024-02166-1

**Published:** 2024-09-11

**Authors:** Xinli Li, Guoqiang Yang, Lihong Ma, Bingxi Tang, Tao Tao

**Affiliations:** 1 Department of Clinical Laboratory, Zibo Central Hospital, Zibo, China; 2 Department of Gastroenterology, Zibo Central Hospital, Zibo, China

**Keywords:** Methylation, Cancer metabolism

## Abstract

Emerging evidence has shown that ferroptosis and antitumor immunity response of T lymphocytes play critical roles in multiple malignancies, including gastric cancer (GC). Here, the present research aims to reveal the function of novel N^6^-methyladenosine (m^6^A) methyltransferase METTL5 on GC immune microenvironment. Clinically, elevated METTL5 was negatively correlated to the prognosis of GC patients. METTL5 high-expression repressed the Fe^2+^ accumulation and ferroptosis to promote the GC immune evasion escaping from activated PBMCs’ killing effect. Mechanistically, upregulation of METTL5 promoted NRF2 mRNA stability, thereby inactivating the ferroptosis and repressing PBMCs’ cells antitumor immunity. One valuable finding is that ferroptosis inhibitor (Ferrostatin-1, Fer-1) could reduce the antitumor immunity of cocultured PBMCs. In other words, the increase of ferroptosis might contribute to the anti-tumor efficacy of immunotherapy. Further study revealed that m^6^A reader IGF2BP1 mediated the stability of NRF2 mRNA via METTL5/m^6^A/NRF2 axis. Collectively, these results demonstrate that METTL5 functions as an oncogene in GC immune microenvironment, and highlights a critical role in T lymphocytes’ antitumor immunity.

## Introduction

Since gastric cancer (GC) is one of the main causes of cancer-related mortality, a lot of research is being done on the identification and application of various biomarkers for prognosis prediction [1]. GC ranks third in terms of cancer-related mortality and is the fifth most common type of cancer to be diagnosed [2]. The majority of patients receive an advanced diagnosis and have a dismal prognosis due to the lack of obvious early symptoms of GC. According to recent data, East Asian nations appear to have a notably high incidence of GC. For higher effective GC treatment and lower mortality, it is crucial to identify the carcinogenic molecules associated with GC.

N^6^-methyladenosine (m^6^A), a common chemical modification in eukaryotic mRNAs, plays crucial roles in cancer advancement. Methyltransferases like METTL3/14/16 (also called “writers”), demethylases like FTO/ALKBH5 (also called “erasers”), and m^6^A binding proteins like IGF2BP1/2/3 and YTHDF1/2/3 (also called “readers”) are involved in the m^6^A methylation process. Research has indicated that the novel enzyme METTL5 functions as an oncogenic factor in hepatocellular carcinoma [3], intrahepatic cholangiocarcinoma [4]. However, little is known about how METTL5-mediated m^6^A modification affects GC tumorigenesis.

In the tumor microenvironment, the purpose of immunotherapy is to enhance the effector function of CD8^+^ T cells [5]. Activated CD8^+^ T cells resulting from cancer immunotherapy primarily eliminate tumors by causing cell death via the Fas-Fas ligand and perforin-granzyme processes. Ferroptosis is one type of cell death, which is distinct from other iron-dependent lipid peroxide accumulation or apoptosis. According to growing findings, ferroptosis may be related to tumor immunity response. It is unknown if and how ferroptosis functions in cancer immunotherapy and CD8^+^ T cell immunity [6].

METTL5 is a novel m^6^A modification methyltransferase that frequently participated in the tumor cancerous progress [7]. However, the role of METTL5-mediated m^6^A modification in GC antitumor immunity and ferroptosis remain largely unexplored [8]. The NRF2 (nuclear factor erythroid 2-related factor 2) is a classical transcription factor that frequently hyperactivated in human cancer, including GC [9, 10]. Here, this research investigated the function of METTL5 on GC cells’ ferroptosis and the antitumor immunity of activated PBMCs’ cells. The ferroptosis might be closely correlated to the antitumor immunity of activated PBMCs. Ferroptosis inhibitor (Ferrostatin-1, Fer-1) could reduce the antitumor immunity of cocultured PBMCs. The findings also revealed that m^6^A reader IGF2BP1 mediated the stability of NRF2 mRNA via METTL5/m^6^A/NRF2 axis. Collectively, these results demonstrate that METTL5 functions as an oncogene in GC immune microenvironment, and highlights a critical role in T lymphocytes’ antitumor immunity.

## Results

### m^6^A methyltransferase METTL5 and NRF2 up-regulated in GC

**Fig. 1:**
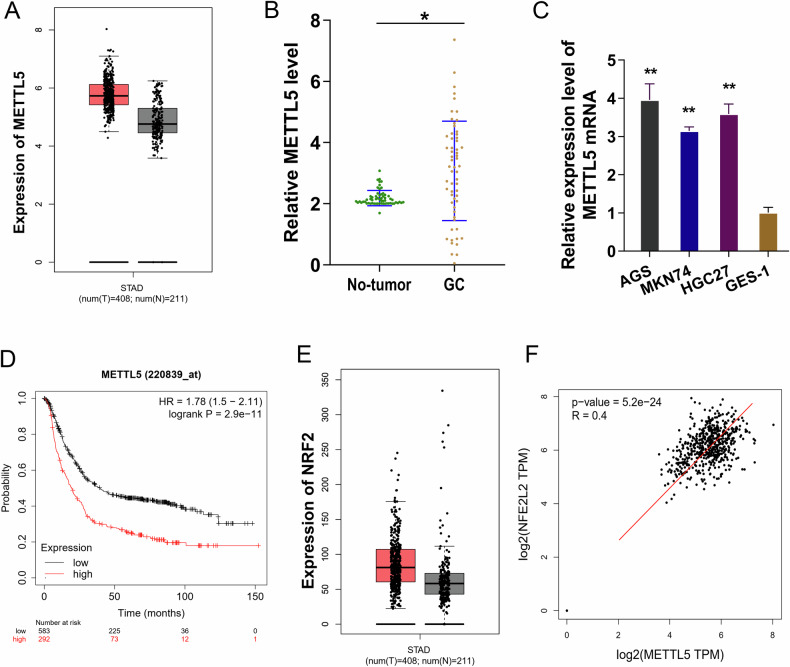
m^6^A methyltransferase METTL5 up-regulated in GC. **A** In GC (STAD, Stomach adenocarcinoma) samples, METTL5 showed an increased expression as comparing to non-tumor samples. **B** The METTL5 levels in GC clinical samples and the para-tumor samples. **C** The METTL5 levels in normal stomach cells (GES-1, human gastric epithelial cells) and GC cells (MKN74, HGC27, AGS) were tested by RT-PCR. **D** The prognosis was tested by Kaplan Meier plotter (http://kmplot.com/analysis/). The group with higher METTL5 level had a poorer survival rate in the prognosis analysis. **E** In GC (STAD, Stomach adenocarcinoma) samples, NRF2 showed an increased expression as comparing to non-tumor samples. **F** GEPIA database (http://gepia.cancer-pku.cn/index.html) showed the positive correlation between the NRF2 enrichment and METTL5 in the correlation analysis. *p < 0.05; **p < 0.01.

Currently, METTL5 is found to be involved in a number of human malignancies, which has been established throughout the preliminary literatures [11]. Here, we also discovered that, in GC samples, METTL5 showed an increased expression (Fig. 1A). When comparing the GC clinical samples, METTL5 level was increased comparing to the para-tumor samples (Fig. 1B). Consistently, the GC cells had a higher amount of METTL5 expression (Fig. 1C). The group with a higher METTL5 level had a poorer survival rate in the prognosis analysis, indicating that METTL5 was a survival risk factor for GC (Fig. 1D). Here, we also discovered that GC samples had increased NRF2 expression (Fig. 1E). The GEPIA database also showed a positive correlation between the NRF2 enrichment and METTL5 in correlation analysis (Fig. 1F). Taken together, these data indicated that m^6^A methyltransferase METTL5 and NRF2 up-regulated in GC.

### METTL5 mitigated the erastin-induced proliferation inhibition of GC cells

**Fig. 2:**
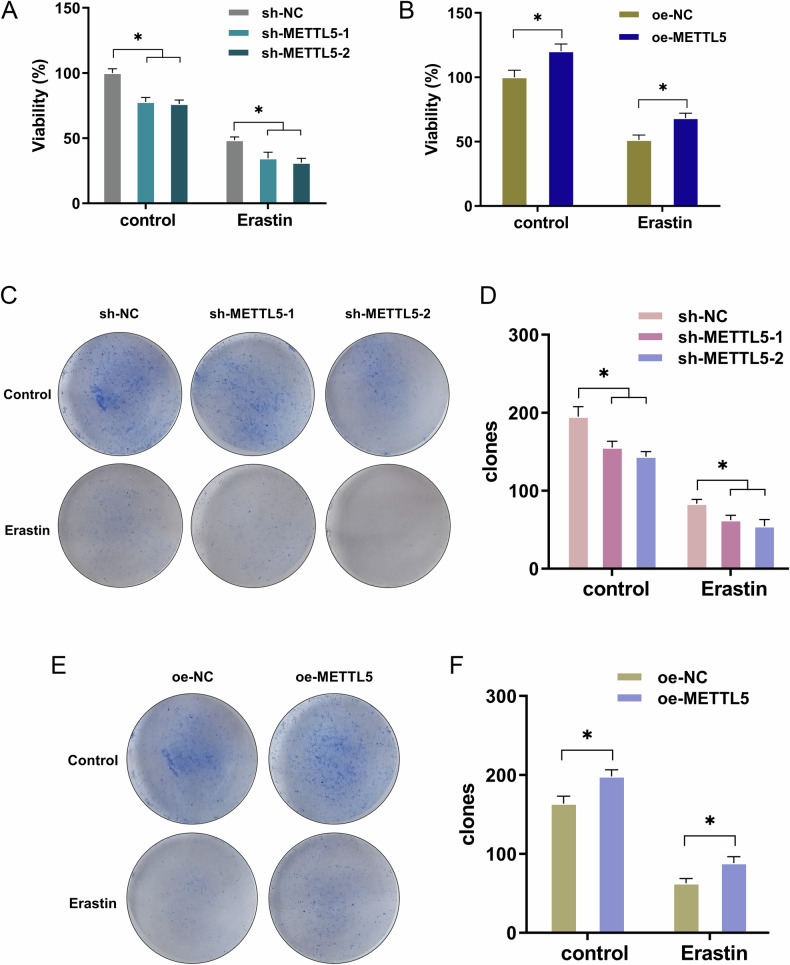
METTL5 mitigated the erastin-induced proliferation inhibition of GC cells. **A**, **B** CCK-8 assays are conducted to clarify the effect of METTL5 on the ferroptosis agonist Erastin (10 μmol/L) induced ferroptosis in AGS (with METTL5 silencing, sh-METTL5-1/2, sh-NC) and MKN-45 (with METTL5 overexpression, oe-METTL5, oe-NC) cells. **C**–**F** The colony formation analysis illustrated the role of erastin-triggered ferroptosis on clone number of GC cells. AGS cells were transfected with METTL5 silencing, and MKN-45 cells were transfected with METTL5 overexpression. Results are evaluated using the two-tailed Student’s t-test. *p < 0.05.

For the ferroptosis-related proliferation analysis, ferroptosis agonist erastin (10 μmol/L) was utilized to treat GC cells (AGS, MKN-45). Proliferation assay was performed to test the cellular viability of GC cells treated with erastin. Results indicated that the ferroptosis induced by erastin could reduce the viability of GC cells. Moreover, the silencing of METTL5 decreased the viability of GC cells (Fig. 2A), and overexpression of METTL5 promoted the viability (Fig. 2B). Furthermore, the colony formation analysis also illustrated that the erastin-triggered ferroptosis impaired clone number of GC cells. In more depth, the silencing of METTL5 decreased the clone number of GC cells (Figs. 2C, D), and overexpression of METTL5 promoted the clone number (Figs. 2E, F). Conclusively, the results obtained from this study affirmed that METTL5 mitigated the erastin-induced proliferation inhibition of GC cells.

### METTL5 impaired the ferroptosis of GC cells

**Fig. 3:**
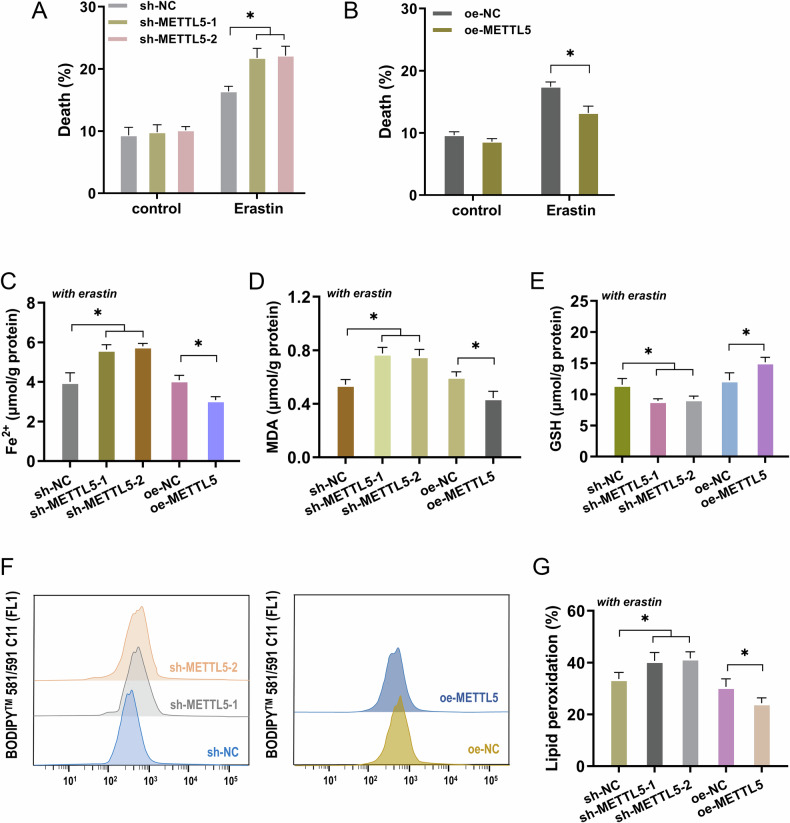
METTL5 impaired the ferroptosis of GC cells. **A**, **B** The cell death was tested by flow cytometry in GC cells with erastin administration (10 μmol/L). AGS cells were transfected with METTL5 silencing (sh-METTL5-1/2), and MKN-45 cells were transfected with METTL5 overexpression (oe-METTL5). **C** Fe^2+^ concentration was analyzed by iron colorimetric assay kit with erastin administration. **D** Malondialdehyde (MDA) was tested by Lipid Peroxidation MDA Assay Kit for quantitative detection with erastin administration. **E** Cellular glutathione (GSH)/oxidized GSH (GSSG) ratio was measured by GSH and GSSG assay kit with erastin administration. **F**, **G** The lipid peroxidation was tested by Flow cytometry in GC cells with erastin administration. *p < 0.05; **p < 0.01.

Ferroptosis is known as the accumulation of lipid peroxides (lipid-ROS) dependent on iron, which results in fatal cellular damage. Both the upstream (ROS production) and downstream (ferroptosis execution) phases of the process can be distinguished. Data from this study indicated that erastin administration resulted in an increase in cell death when ferroptosis was induced (Fig. 3A, B). Concretely, the silencing of METTL5 accelerated the cell death and the overexpression of METTL5 mitigated the cell death of GC cells. To test how METTL5 regulated the ferroptosis of GC cells, the ferroptosis-related features (Fe^2+^, MDA, GSH, and lipid peroxidation) were detected with erastin administration. Results demonstrated that silencing of METTL5 accelerated the Fe^2+^ (Fig. 3C), MDA (Fig. 3D), and lipid peroxidation (Fig. 3F, G) accumulation, and repressed the GSH production (Fig. 3E) under erastin administration. Moreover, overexpression of METTL5 alleviated the Fe^2+^, MDA, and lipid peroxidation accumulation, and up-regulated the GSH production. Taken together, the outcomes of this part demonstrated that METTL5 impaired the ferroptosis of GC cells.

### METTL5 ameliorated the antitumor immunity of activated PBMCs to GC cells in vitro

**Fig. 4:**
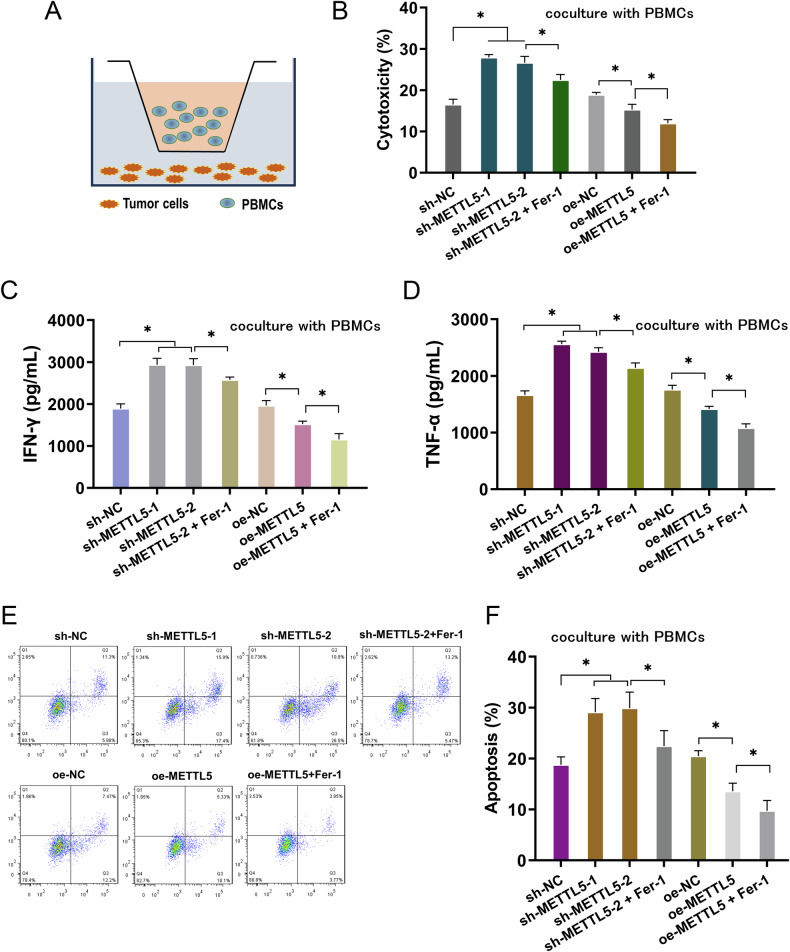
METTL5 and ferroptosis inhibitor (Fer-1) ameliorated the antitumor immunity of activated PBMCs to GC cells in vitro. **A** The coculture system was constructed using activated PBMCs and GC cells. **B** The cytotoxicity activity of activated PBMCs was detected by LDH release analysis in the coculture of GC cells and PBMCs. GC cells were transfected with METTL5 silencing (sh-METTL5-1/2) and overexpression plasmids (oe-METTL5). Ferroptosis inhibitor ferrostatin-1 (Fer-1, 1 μM, 6 h) was administrated to the coculture. **C** Cytokines IFN-γ and **D** TNF-α secreted by activated PBMCs were tested using ELISA kit when cocultured with GC cells. Ferroptosis inhibitor ferrostatin-1 (Fer-1, 1 μM, 6 h) was administrated to the coculture. **E**, **F** The apoptosis of cocultured GC cells was tested using flow cytometry. Ferroptosis inhibitor ferrostatin-1 (Fer-1, 1 μM, 6 h) was administrated. Results are evaluated using the two-tailed Student’s t-test. *p < 0.05; **p < 0.01.

In order to investigate how METTL5 regulate the antitumor immunity of activated PBMCs to GC cells, the coculture system was constructed (Fig. 4A). After incubation with activated PBMCs in the coculture system, the cytotoxicity of PBMCs towards GC cells was measured by LDH release analysis. Data illustrated that the cocultured PBMCs exerted higher cytotoxicity activity when co-cultured with METTL5 silencing transfected GC cells, and lower cytotoxicity activity in METTL5 overexpression transfected GC cells (Fig. 4B). Moreover, the Ferroptosis inhibitor administration, Ferrostatin-1 (Fer-1), could reduce the cytotoxicity activity of PBMCs, suggesting the immunosuppression of ferroptosis inhibitor on GC. The cytokines (IFN-γ, TNF-α) secreted by activated PBMCs were tested and data indicated that the cocultured PBMCs exerted higher cytokines (IFN-γ, TNF-α) when cocultured with METTL5 silencing GC cells (Fig. 4C, D), and exerted lower cytokines when cocultured with METTL5 overexpression GC cells. The Fer-1 administration both reduced the cytokines level. For the apoptosis of cocultured GC cells, the apoptosis was increased when METTL5 was silenced, and reduced when METTL5 was overexpressed (Fig. 4E, F). Besides, Fer-1 administration decreased the apoptosis of GC cells. Taken together, this finding demonstrated that METTL5 ameliorated the antitumor immunity of activated PBMCs to GC cells in vitro.

### METTL5 enhanced NRF2 stability through m^6^A binding

**Fig. 5:**
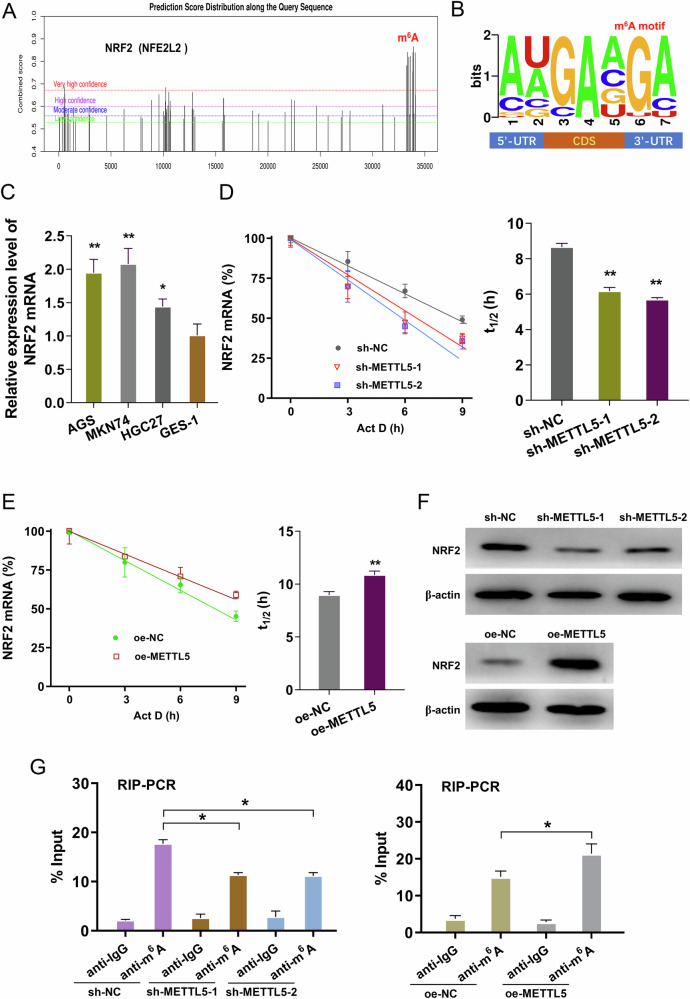
METTL5 enhanced NRF2 stability through m^6^A binding. **A** SRAMP (http://www.cuilab.cn/sramp) demonstrated that there was remarkable m^6^A site on the NRF2 (NFE2L2 gene). **B** The m^6^A motif on NRF2 was focused on ‘GA’ nucleotides. **C** NRF2 mRNA expression was tested using RT-PCR. **D**, **E** RNA decay analysis was performed to test the half life time (t_1/2_) of NRF2 mRNA with METTL5 silencing or METTL5 overexpression. **F** NRF2 protein level was detected in GC cells with METTL5 silencing or METTL5 overexpression. **G** RIP-PCR was performed to detect the immunoprecipitated NRF2 RNA using anti-m^6^A antibody. RNA samples were extracted from GC cells. Results are evaluated using the two-tailed Student’s t-test. *p < 0.05; **p < 0.01.

The nuclear factor erythroid 2-related factor 2 (NRF2) is a classical transcription factor that is frequently hyperactivated in human cancer, including GC. Given the positive connection of METTL5 and NRF2, the assays further tried to investigate the role of METTL5 on GC ferroptosis and antitumor immunity through NRF2. Firstly, there was remarkable m^6^A site on the NRF2 (NFE2L2 gene) (Fig. 5A). The m^6^A motif on NRF2 was focused on ‘GA’ nucleotides (Fig. 5B). GC cells had a high amount of NRF2 mRNA expression (Fig. 5C). RNA decay analysis revealed that METTL5 silencing repressed the half life time (t_1/2_) of NRF2 mRNA (Fig. 5D**)**, and METTL5 overexpression promoted the half life time (t_1/2_) of NRF2 mRNA (Fig. 5E**)**. Analogously, the METTL5 silencing repressed the NRF2 protein level, and METTL5 overexpression promoted NRF2 protein level (Fig. 5F**)**. RIP-PCR analysis revealed that METTL5 silencing inhibited the immunoprecipitated NRF2 using anti-m^6^A antibody, and METTL5 overexpression facilitated the immunoprecipitated NRF2 (Fig. 5G**)**. In summary, the data of these assays demonstrated that METTL5 enhanced NRF2 stability through m^6^A binding.

### IGF2BP1 regulated NRF2 mRNA fate through the m^6^A modified site

**Fig. 6:**
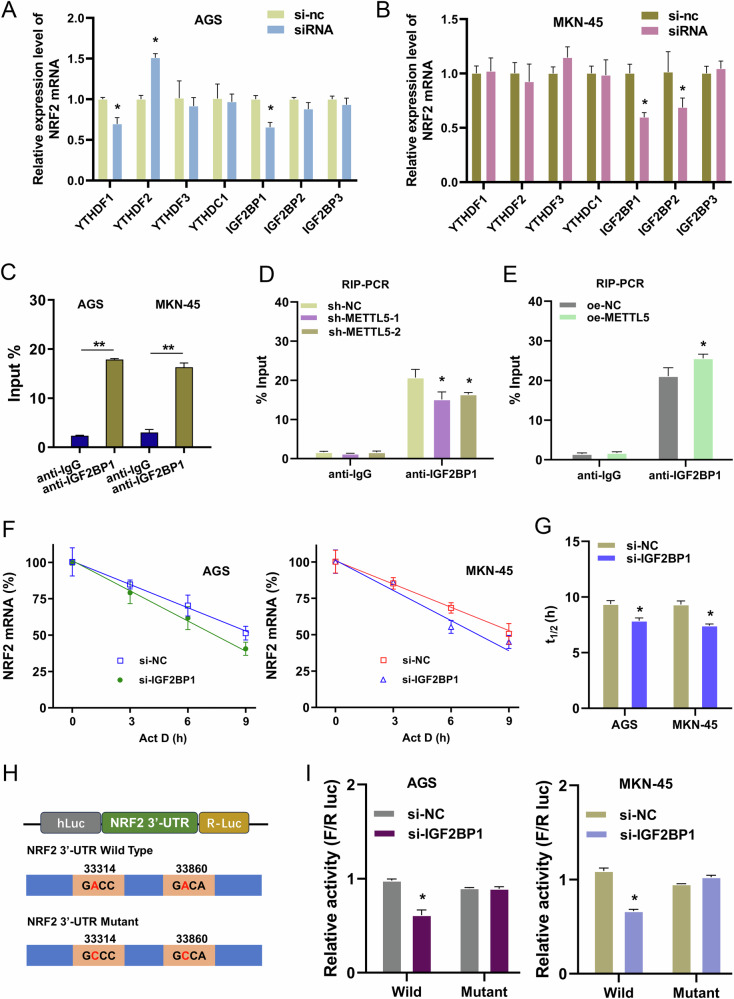
IGF2BP1 regulated NRF2 mRNA fate through the m^6^A modified site. **A**, **B** The NRF2 mRNA was detected using RT-PCR in GC cells (AGS, MKN-45) transfected with siRNA targeting several m^6^A readers. **C** RIP-PCR was performed in GC cells (AGS, MKN-45) using anti-IGF2BP1/anti-IgG to detect the NRF2 mRNA level in immunoprecipitation. **D**, **E** The RIP-PCR was performed in GC cells (AGS, MKN-45) using anti-IGF2BP1/anti-IgG to detect the NRF2 mRNA with METTL5 silencing or overexpression. AGS cell was transfected with sh-METTL5-1/2, and MKN-45 cell was transfected with oe-METTL5. **F**, **G** RNA decay analysis revealed the half life time (t_1/2_) of NRF2 mRNA in GC cells with IGF2BP1 silencing (si-IGF2BP1). **H** The luciferase reporters were inserted either the wild-type (WT) or mutant (Mut) sequences. **I** The luciferase activity was detected when co-transfected with NRF2-WT/Mut vectors and IGF2BP1 silencing (si-IGF2BP1). Results are evaluated using the two-tailed Student’s t-test. *p < 0.05; **p < 0.01.

To uncover the deep mechanism how the m^6^A modification affect the NRF2 mRNA fate, the alternative m^6^A readers were tested in GC cells. Results indicated that there were several m^6^A readers altered the NRF2 mRNA levels (Fig. 6A). From these readers, IGF2BP1 exhibited a significant altered effect on the NRF2 mRNA in both cell lines (Fig. 6B). Thus, the m^6^A reader IGF2BP1 was considered as the effector to regulate NRF2 mRNA fate. In following analysis, the RIP-PCR assay indicated that IGF2BP1 significantly interacted with NRF2 mRNA in GC cells (Fig. 6C). Moreover, METTL5 silencing reduced the interaction within IGF2BP1 and NRF2 mRNA, and METTL5 overexpression promoted the interaction within IGF2BP1 and NRF2 mRNA (Fig. 6D. E). RNA decay analysis revealed that IGF2BP1 silencing (si-IGF2BP1) repressed the half-life time (t_1/2_) of NRF2 mRNA (Fig. 6F, G). Another luciferase reporter assay was designed to show the impact of m^6^A on the fate of NRF2 mRNA. The luciferase reporters were inserted either the wild-type (WT) or mutant (Mut) sequences (Fig. 6H). Cells transfected with the NRF2-WT plasmid tended to exhibit lower luciferase activity when IGF2BP1 was silenced (Fig. 6I). Thus, the data of luciferase analysis indicated that IGF2BP1 regulated the NRF2 mRNA fate via m^6^A modified sites. In conclusion, we could draw a conclusion that IGF2BP1 regulated NRF2 mRNA fate through the m^6^A modification.

### METTL5 targeted IGF2BP1/m^6^A/NRF2 axis to regulate GC ferroptosis and activated PBMCs antitumor immunity

**Fig. 7:**
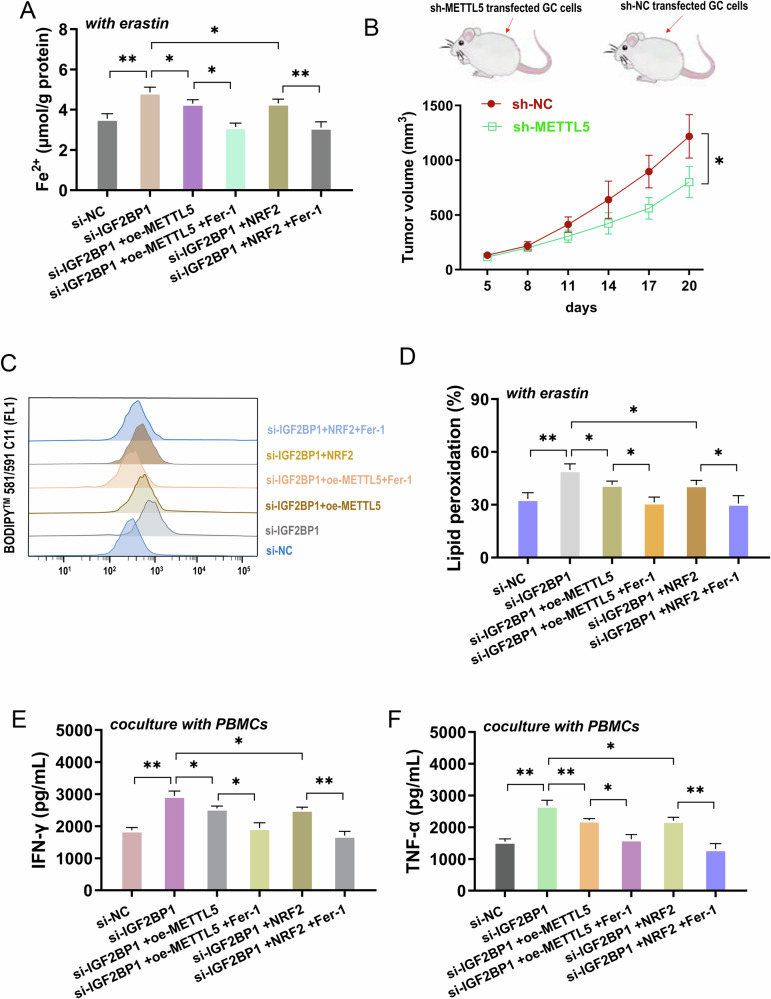
METTL5 targeted IGF2BP1/m^6^A/NRF2 axis to regulate GC ferroptosis and activated PBMCs antitumor immunity. **A** Fe^2+^ concentration was analyzed in GC (AGS) cells transfected with IGF2BP1 silencing (si-IGF2BP1), METTL5 overexpression (oe-METTL5) and NRF2 overexpression (NRF2). Ferroptosis inhibitor (Ferrostatin-1, Fer-1) was administrated to cells. **B** In the in vivo mice xenograft assay, the METTL5 silencing (sh-METTL5) transfected AGS cells were injected to mice. **C**, **D** The lipid peroxidation was tested by Flow cytometry in GC cells. **E** Cytokines IFN-γ and (**F**) TNF-α secreted by activated PBMCs were tested using ELISA kit when cocultured with GC cells. Ferroptosis inhibitor ferrostatin-1 (Fer-1, 1 μM, 6 h) was administrated to the coculture. *p < 0.05; **p < 0.01.

**Fig. 8:**
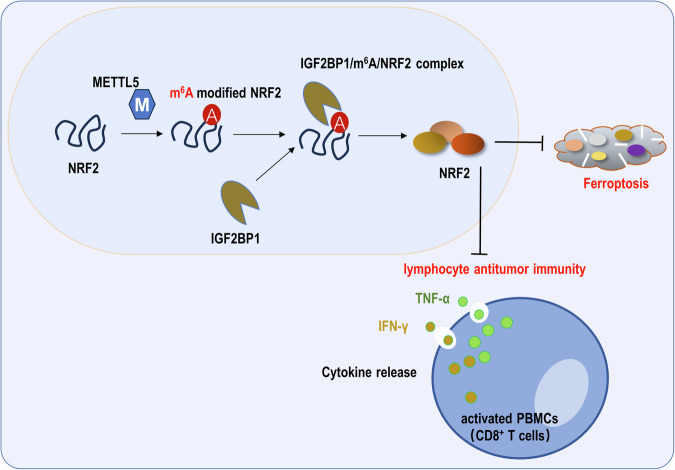
m^6^A writer METTL5 targets NRF2 to ameliorate GC cells ferroptosis and represses activated PBMCs (CD8^+^ T cells) antitumor immunity.

Given that the previous data revealed the important role of METTL5 on GC ferroptosis and activated PBMCs antitumor immunity, the rescue assays were performed to verify the following IGF2BP1/m^6^A/NRF2 axis. Results demonstrated that IGF2BP1 silencing accelerated the Fe^2+^ (Fig. 7A), lipid peroxidation (Fig. 7C, D) accumulation, and cytokines (IFN-γ, TNF-α) (Fig. 7E, F) secreted by activated PBMCs. Moreover, METTL5 overexpression (oe-METTL5) or NRF2 overexpression (NRF2) co-transfection repressed the Fe^2+^, lipid peroxidation accumulation, and cytokines (IFN-γ, TNF-α). Moreover, the Ferroptosis inhibitor administration, Ferrostatin-1 (Fer-1), could reduce the Fe^2+^, lipid peroxidation accumulation, and cytokines (IFN-γ, TNF-α). Besides, in the in vivo mice xenograft assay, the METTL5 silencing reduced the tumor growth (volume) (Fig. 7B). In summary, these data revealed that METTL5 targeted IGF2BP1/m^6^A/NRF2 axis to regulate GC ferroptosis and activated PBMCs antitumor immunity. As shown in Fig. 8, this present study revealed that METTL5 functions as an oncogene in GC immune microenvironment by targeting ferroptosis and PBMCs’ cells antitumor immunity via IGF2BP1/m6A/NRF2 axis, which highlights a critical role in CD8^+^ T lymphocytes’ antitumor immunity.

## Discussion

The tumor immunotherapy can restore or enhance the effector function of cytotoxic T cells (CD8^+^ T cells) in the tumor microenvironment [12, 13]. Lymphocyte (in especial CD8^+^ T cells) activated by tumor immunotherapy mainly eliminate tumors by secreting cytokines, producing perforin/granzyme and inducing cell death through the Fas-Fas ligand pathway. Besides, ferroptosis is a form of cell death distinct from apoptosis and is the result of iron-dependent accumulation of lipid peroxide [14]. In our present research, we performed these assays to investigate the function of m^6^A modification on GC antitumor immunity and ferroptosis.

METTL5 is a novel m^6^A modification methyltransferase that frequently participated in the tumor cancerous progress. However, the role of METTL5-mediated m^6^A modification in GC antitumor immunity and ferroptosis remain largely unexplored. Here, our research found that METTL5 showed an increased expression in GC clinical samples and GC cells. Clinically, the patients with a higher METTL5 level had a poorer survival rate in the prognosis analysis, indicating the risk factor of METTL5 for GC. In functional analysis of METTL5 on GC cells in vitro, the results found that METTL5 mitigated the ferroptosis agonist erastin-induced proliferation inhibition of GC cells. Moreover, METTL5 impaired the ferroptosis-related features (Fe^2+^, MDA, GSH, and lipid peroxidation), suggesting that METTL5 repressed the ferroptosis of GC cells.

Our study not only investigated the GC cells’ ferroptosis, but also focused on exploring its relationship with tumor immunity. Presently, the role of m^6^A modification in GC antitumor immunity and ferroptosis remain largely unexplored. In our findings, we found that METTL5 and ferroptosis inhibitor (Ferrostatin-1, Fer-1) both ameliorated the antitumor immunity of activated PBMCs to GC cells in vitro. In this study, the activated PBMCs was utilized to simulate the CD8^+^ T cells in the coculture system with GC cells. Therefore, METTL5 and ferroptosis inhibitor Fer-1 could reduce the antitumor immunity of cocultured PBMCs. In other words, the increase of ferroptosis might contribute to the anti-tumor efficacy of immunotherapy.

Emerging evidence suggests that ferroptosis may be related to the tumor immune microenvironment. Cell death caused by excessive accumulation of metal ions has received increasing attention, such as cuproptosis [15, 16] and ferroptosis. Whether and how ferroptosis participates in T cell immunity and tumor immunotherapy is of great potential research value. Some studies have shown that CD8^+^ T cells activated by immunotherapy enhance ferroptosis-specific lipid peroxidation in tumor cells, and the increase of ferroptosis contributes to the anti-tumor efficacy of immunotherapy. The ferroptosis inhibitor administration (Ferrostatin-1, Fer-1) could reduce the cytotoxicity activity of activated PBMCs in coculture, suggesting the immunosuppression of Fer-1 on GC. Thus, these data suggest that ferroptosis was closely correlated to the tumor immunology.

The further research revealed that METTL5 high-expression repressed the Fe^2+^ accumulation and ferroptosis to promote the immune evasion escaping from activated PBMCs’ killing effect. Mechanistically, upregulation of METTL5 promoted NRF2 mRNA stability, thereby inactivating the ferroptosis and PBMCs’ cells antitumor immunity. In this progression, m^6^A reader IGF2BP1 mediated the stability of NRF2 mRNA via METTL5/m^6^A/NRF2 axis. Nuclear factor erythroid 2-related factor 2 (NRF2) has frequently been in connection with ferroptosis [17], including head and neck cancer [18], GC [19, 20], and breast cancer [21, 22].

Collectively, these results demonstrate that METTL5 functions as an oncogene in GC immune microenvironment and ferroptosis phenotype. The ferroptosis is closely correlated to the antitumor immunity of T lymphocytes in GC. Furthermore, m^6^A reader IGF2BP1 mediated the stability of NRF2 mRNA via METTL5/m^6^A/NRF2 axis. Collectively, these results demonstrate the roles of METTL5 on GC tumorigenesis, and highlight a critical role in T lymphocytes’ antitumor immunity.

## Materials and methods

### Clinical specimens

From Zibo Central Hospital, human GC tissues as well as a pair of adjacent normal tissues were acquired. Following surgical resection, the tumor and any mucosal tissue that was within 3–5 cm of the tumor’s edge were cut into species, liquid nitrogen was used to snap-freeze them, and they were kept at -80 °C. The assay was approved by Ethics Committee of Zibo Central Hospital.

### Cell lines of GC and transfection

Normal stomach cells (GES-1, human gastric epithelial cells) and GC cells (MKN74, HGC27, AGS) were purchased from ATCC (Maryland, USA) and maintained in Dulbecco’s Modified Eagle’s Medium (Gibco) supplied with 10% FBS (BI, Israel) at 37 °C in humid environment containing 5% CO_2_. The transfection was performed using lipofectamine 2000 (Invitrogen) according to the manufacturer’s instruction protocols. For the ferroptosis analysis, erastin (10 μmol/L) was utilized to treat GC cells.

For the overexpression transfection, the full-length cDNA of human METTL5 (GenBank accession no. NM_001293186.2), NRF2 (GenBank accession no. NM_001145412.3) were amplified from their DNA template using standard PCR-based clone. For silencing, lentivirus vector containing METTL5 shRNA was purchased from OBiOc (Shanghai, China). To construct a lentivirus-mediated silencing vector, shRNA sequences targeting human METTL5 were cloned into shRNA vectors. After lentivirus transfection, GC cells were treated with 2 mg/mL puromycin to select the infected cells. The siRNA targeting (si-IGF2BP1, 15 nM) was transfected into GC cells using Lipofectamine RNA iMAX reagent (Invitrogen, cat. MAN0007825) according to manufacturer’s instruction for gene silencing.

### RNA extraction and qPCR and western blot

In accordance with the manufacturer’s instructions, total RNA samples were isolated using Life Technologies’ Trizol Reagent (cat. 15596026). High Capacity cDNA Reverse Transcription Kit (Applied Biosystems, Darmstadt, Germany) was used to reversely transcribe total RNA (1 mg) for the synthesis of cDNA. PCR reaction was conducted using SYBR Green PCR Master Mix (Applied Biosystems) on an ABI7500 Real-time. Endogenous control was β-actin or GAPDH. Each reaction of this analysis was performed three times. Supplementary Table S1 showed the list of sequence-specific primers for the genes that referred. For the western blot assay, the primary antibody (anti-NRF2, 1:1000, Abcam, ab62352) was used to be incubated with the extracted proteins. The quantitative analysis of the intensity of each band was calculated using the ImageJ software.

### Proliferation analysis

The proliferation ability of GC cells was analyzed by CCK-8 and colony formation assay. To quantify cell proliferation, the CCK-8 test kit (Dojindo Japan) was utilized. To put it briefly, 96-well culture plates were used to seed GC cells. Cells were starved in FBS-free media for 12 hours prior to treatment. CCK-8 reagent (10 μl) was added to each well after the appropriate amount of time had been incubated, and the absorbance at 450 nm was measured. Every trial was conducted at least three times. For the colony formation assay, 1000 GC cells per well had undergone stable transfection were cultivated in DMEM-coated 6-well plates. Crystal violet staining solution (1%) was used to color proliferating colonies (cat. E607309, Sangon Biotech). The colonies were measured and captured on camera for counting.

### Iron (Fe^2+^), MDA and glutathione (GSH) analysis

Iron (Fe^2+^) was detected using Iron colorimetric assay kit (Applygen, Beijing, China, cat. E1042) for quantitative detection. The malondialdehyde (MDA) was detected using Lipid Peroxidation MDA Assay Kit (Beyotime Biotechnology, Jiangsu, China, cat. S0131S). Glutathione (GSH) was detected using GSH and GSSG assay kit (Beyotime Biotechnology, cat. S0053).

### Lipid peroxidation and death analysis

Flow cytometry was used to analyze and identify lipid peroxidation and cell death, as previously reported. For lipid peroxidation, GC cells were seeded in six-well plates and incubated with C11- BODIPY (5 μM, cat. D3861, Thermo Fisher) and finally tested by flow cytometric analysis with ID7000™ Spectral Cell Analyzer (Sony Biotechnology). For the death calculation, using the Annexin V-FITC/propidium iodide (PI) apoptosis detection kit (Vazyme) double staining method, the amount of cell death was determined by BD FACSCalibur (BD Biosciences, Franklin Lakes, NJ, USA).

### Activated PBMCs coculture

The blood supply was provided from the white blood cell concentrate of healthy female donors, from which our lab extracted the peripheral blood mononuclear cells (PBMCs). PBMCs were enriched using Ficoll kit (Sigma-Aldrich, USA) in accordance with the supplier’s instructions. PBMCs were activated in media containing anti-CD3 (2 μg/mL), anti-CD28 (3 μg/ml) and IL-2 (200 U/ml, Biolegend, Inc., USA) for 24 h. For cell culture, RPMI medium (Invitrogen) with 10% FBS and 100 U/mL penicillin-streptomycin was used. Activated PBMCs were co-cultured with GC cells (effector: target=5:1) for seven days after activation.

### Cytotoxicity assay

The activated PBMCs mediated cytotoxicity on GC cells was determined by LDH assay. After coculture of activated PBMCs and GC cells for 7 hours, the culture supernatants were collected for lactate dehydrogenase (LDH) release test. using the Cytotoxicity Detection Kit PLUS (cat. 04744926001, Sigma-Aldrich) according to the manufacturer’s instruction. The cytotoxicity was calculated as previous reported [23].

### Cytokines of IFN-γ and TNF-α

The concentrations of cytokines (IFN-γ and TNF-α) secreted from activated PBMCs were measured by IFN-γ (eBioscience, Cat: KHC4021) and TNF-α (eBioscience, Cat: BMS223HS) in accordance with the manufacturer’s guideline.

### RNA stability assay

After transfecting cells with the specified plasmids or shRNAs, GC cells were treated with 5 mg/mL actinomycin D (cat. SBR00013, Sigma-Aldrich). After that, RNAs were extracted and calculated the relative expression of NRF2 mRNA at different times using qRT-PCR.

### RNA-binding immunoprecipitation qPCR

The interaction between METTL5, IG2BP1 and its target NRF2 mRNAs were verified by RNA-binding immunoprecipitation qPCR (RIP-qPCR). The RIP assay was performed according to the instructions. Briefly, 150 mm culture plates were used to cultivate GC cells that at 80% confluence. After a single ice-cold PBS washing, the GC cells were lysed in RNA immunoprecipitation (RIP) buffer, which contained 100 U/mL RNAase inhibitor (cat. B600478, Sangon Biotech) and protease inhibitors. The IP buffer composition was 0.5% NP40, 150 mmol/L KCl, 5 mmol/L EDTA, 25 mmol/L Tris pH 7.4 and 0.5 mmol/L DTT. Then, the cell extract was incubated by Protein A/G magnetic beads (cat. B26101, Bimake) that conjugated with METTL5 antibody or m^6^A-specific antibody for 6 h at 4 °C, as well as the negative control IgG antibody. Lastly, the coprecipitated RNA was extracted and subjected to qPCR calculation.

### MeRIP-qPCR assay

Following the extraction of RNAs from the GC cells, anti-m^6^A antibody (Thermo Fisher, USA) was incubated for six-hours at 4 °C. At room temperature, BSA (2%, Thermo Fisher) was used to wash the RNA for three hours before incubating the RNA-antibody mixture at 4 °C for overnight on Dynabeads (Invitrogen, USA). After elution with SDS buffer, the RNAs were separated, and qPCR analysis was used to measure the levels of m^6^A modification expression on mRNA.

### Luciferase reporter assay

Luc-NRF2-3’UTR and Luc-NRF2-3′UTR-MUT were the dual-luciferase reporter gene vectors of target gene NRF2 and mutants with mutations in the IGF2BP1-binding region, respectively. IGF2BP1 and IGF2BP1-siRNA reporter plasmids were co-transfected into 293 T cells. The cells were lysed 36 hours after transfection and centrifuged for one minute at 12,000 rpm. The Dual-Luciferase® Reporter Assay System (Promega) was then used to measure the amount of luciferase present in the supernatant.

### In vivo mice assay

Approximately 1 × 10^6^ GC cells (AGS, sh-NC, sh-METTL5) per mouse suspended in PBS (100 μl) were injected into the flank of BALB/c nude mice (male, 4–6 weeks old). During the three weeks-day observation, the tumor size = (width^2^ × length × 0.52) was measured by a vernier caliper.

### Quantification and statistical analysis

For the quantification, the statistical analysis was performed using GraphPad Prism 8.0. As indicated, error bar was shown as mean ± SEM or mean ± SD. Significance within groups was determined using unpaired Student two-tailed t-test or One-way analysis. *p* < 0.05; *p* < 0.01 as significant.

## Supplementary information

blot-1

blot-2

supplement Table S1

## Data Availability

The datasets generated during and/or analysed during the current study are available from the corresponding author on reasonable request.
