# Supplementary material for: N6-methyladenosine (m6A) writer METTL5 represses the ferroptosis and antitumor immunity of gastric cancer
Source: Cell Death Discov. 2024 Sep 11;10:402. doi: 10.1038/s41420-024-02166-1 (PMC11390903; doi:10.1038/s41420-024-02166-1)
Supplement: Supplementary file 3 — supplement Table S1 [file 41420_2024_2166_MOESM3_ESM.docx]

**supplementary Table S1**. Primers of qRT-PCR.

|  | Sequences |
| --- | --- |
| METTL5 | F, 5’- AAGGAACTAGAGAGTCGCCTG-3’  R, 5’- GCGGCCTGGTAGGATACTG -3’ |
| NRF2 | F, 5’- TCCAGTCAGAAACCAGTGGAT-3’  R, 5’- GAATGTCTGCGCCAAAAGCTG-3’ |
| sh-METTL5-1 | 5’- GTCATTCGATACAGTAATTAT-3’ |
| sh-METTL5-2 | 5’- TTGCAGCATGTATGCTCTATA-3’ |
| GAPDH | F, 5’- CTGGGCTACACTGAGCACC -3’  R, 5’- AAGTGGTCGTTGAGGGCAATG -3’ |
